# Motif-All: discovering all phosphorylation motifs

**DOI:** 10.1186/1471-2105-12-S1-S22

**Published:** 2011-02-15

**Authors:** Zengyou He, Can Yang, Guangyu Guo, Ning Li, Weichuan Yu

**Affiliations:** 1 School of Software, Dalian University of Technology, Dalian, China; 2 Laboratory for Bioinformatics and Computational Biology, Department of Electronic and Computer Engineering, The Hong Kong University of Science and Technology, Hong Kong, China; 3 Department of Biology, The Hong Kong University of Science and Technology, Hong Kong, China

## Abstract

**Background:**

Phosphorylation motifs represent common patterns around the phosphorylation site. The discovery of such kinds of motifs reveals the underlying regulation mechanism and facilitates the prediction of unknown phosphorylation event. To date, people have gathered large amounts of phosphorylation data, making it possible to perform substrate-driven motif discovery using data mining techniques.

**Results:**

We describe an algorithm called Motif-All that is able to efficiently identify all statistically significant motifs. The proposed method explores a support constraint to reduce search space and avoid generating random artifacts. As the number of phosphorylated peptides are far less than that of unphosphorylated ones, we divide the mining process into two stages: The first step generates candidates from the set of phosphorylated sequences using only support constraint and the second step tests the statistical significance of each candidate using the odds ratio derived from the whole data set. Experimental results on real data show that Motif-All outperforms current algorithms in terms of both effectiveness and efficiency.

**Conclusions:**

Motif-All is a useful tool for discovering statistically significant phosphorylation motifs. Source codes and data sets are available at: http://bioinformatics.ust.hk/MotifAll.rar.

## Background

Protein phosphorylation is an essential post-translational modification event for the regulation and maintenance of most biological processes. Recent advances in high-throughput methods such as tandem mass spectrometry enable rapid and direct discovery of hundreds of phosphorylation sites in a single experiment [1]. The availability of large amounts of phosphorylation sites makes it possible to perform phosphorylation motif finding using data mining techniques.

According to [2] and [3], phosphorylation motif discovery is defined as finding a set of motifs that appear more often in the set of phosphorylated peptides *P* than in the set of unphosphorylated peptides *N*. That means each phosphorylation motif is “over-expressed” in *P*. Here all peptides have the fixed length *L* and they are aligned on the phosphorylated residue. We often call *P* the foreground set and *N* the background set.

The discovery of phosphorylation motifs is computationally challenging. Suppose a motif resides in the peptides of length *L*. This motif is a consensus sequence (including the phosphorylation site) that consists of conserved positions and wild-card positions that can match any residue. The number of all possible phosphorylation motifs is then 21^(^*^L^*^–1)^ – 1. Though the length *L* is usually fixed to be a small value (e.g., 13) in previous studies [2,3], it is still infeasible to perform exhaustive search to check all potential phosphorylation motifs. Besides, it is also unclear which metric is more suitable to measure the statistical significance of the motif.

To efficiently find phosphorylation motifs, two heuristic methods have been proposed. The Motif-X method [2] is a greedy algorithm that reports motifs in an iterative manner. In each round, one most statistically significant motif is detected. The peptides matching the motif identified in the first round are removed prior to the second round of searching. This procedure repeats a number of rounds until no significant motifs can be found. The MoDL method [3] is an optimization-based algorithm, which formulates the motif-finding problem as the minimization of description length. In other words, MoDL tries to optimize the expressiveness of a set of motifs rather than quantifying the significance of a single motif. However, both Motif-X and MoDL can only find a small subset of phosphorylation motifs. They cannot guarantee to find all statistically significant motifs so that some important phosphorylation patterns remain unknown to biologists. Furthermore, Motif-X is limited to discover non-overlap motifs while MoDL unusually reports at most three motifs.

In this paper, we present a new algorithm called Motif-All for the discovery of all statistically significant phosphorylation motifs. Motif-All uses the odds ratio to quantify the over-expressiveness of each motif in the set of phosphorylated peptides against the background set. To avoid exhaustive search, we impose a support constraint for each motif on the set of phosphorylated peptides. The use of support constraint enables us to borrow ideas from association rule mining [4] to generate and prune candidates in a level-wise manner before calculating the odds ratio.

To demonstrate the superiority of Motif-All method, we conduct experimental studies using the PhosPhAt database 3.0 of Arabidopsis phosphorylation sites [5,6]. Motif-All performs better than Motif-X and MoDL in finding more significant phosphorylation motifs. Furthermore, it is very fast and is able to finish the mining of large data sets within a reasonable time period.

The rest of the paper is organized as follows: Section 2 presents the Motif-All algorithm. Section 3 shows the experimental results. Section 4 gives some discussions and Section 5 concludes the paper.

## Methods

There are two critical issues in phosphorylation motif finding. The first is how to measure the over-expressiveness of each motif. The second is how to perform the search in an efficient manner. In the proposed Motif-All method, we use odds ratio to evaluate if one candidate motif is over-expressed. To search efficiently, we use the following strategies to improve the running efficiency of Motif-All.

• We impose a *support* constraint on each candidate motif. Here the support for a motif is defined as the percentage of phosphorylated peptides that match this motif. The notation of support is widely used in association rule mining [4]. One motif is said to be *frequent* if its support is no less than a given threshold. The aim of introducing support constraint is two-fold: on the one hand, it can filter out non-frequent motifs that may correspond to random artifacts; on the other hand, it makes it possible to generate and prune motifs in a level-wise manner so as to avoid brute-force search.

• We divide the mining process into two stages. In the first stage, we perform frequent motif finding using only the data set *P* since the number of phosphorylated peptides is much smaller than that of unphosphorylated ones. In the second stage, we collect support information using the data set *N* to calculate the statistical significance of those candidate motifs from the first stage. We will report all phosphorylation motifs whose *p*-values are no larger than a user-specified significance threshold.

### Odds ratio and statistical significance score

The odds of an event is the probability that this event occurs divided by the probability that it does not occur. The odds ratio is defined as the ratio of the odds of an event in one group to the odds in the complementary group [7].

In the context of phosphorylation motif discovery, the first group corresponds to the set of phosphorylated peptides *P* and the second group is the set of unphosphorylated peptides *N.* For a given motif *m*, we can construct a contingency table as shown in Table 1. In this table, *c*_00_, *c*_01_, *c*_10_ and *c*_11_ are non-negative “cell counts” and  denotes that the motif *m* doesn’t exist. Then, the calculation of odds ratio reduces to:(1)

**Table 1:** A contingency table for a phosphorylation motif

	*m*	**
***P***	*c*_00_	*c*_01_
*N*	*c*_10_	*c*_11_

An odds ratio of 1 means that the target motif is equally likely to exist in both *P* and *N*. An odds ratio greater than 1 indicates that this motif is more likely to appear in the set *P.*

To conduct statistical inference, one approach is to use large sample approximations to the sampling distribution of the log odds ratio. More precisely, the sample log odds ratio is:(2)

and the standard error for the log odds ratio is approximately:(3)

Then, the *z*-value

*Z*(*m*) = *LOR*(*m*)/*SE*(*m*), (4)

follows a standard normal distribution.

Finally, we can calculate the *p*-value to assess the statistical significance of each motif.

### Frequent motif mining

Given a set of phosphorylated peptides *P* and a user-specified support threshold *s,* the objective of frequent motif finding is to discover all motifs whose supports are no less than *s.* The support for a motif is the percentage of phosphorylated peptides in *P* that match this motif. In other words, a motif *m* is frequent if and only if its cell count *c*_00_ in Table 1 satisfies *c*_00_ ≥ *|P|s* with *|* • *|* denoting the size of a set. Note that a similar occurrence constraint is utilized in the Motif-X algorithm [2], which represents the minimal number of phosphorylated peptides needed to match the residue/position pair in its greedy search procedure. There are two fundamental differences between our support constraint and their occurrence constraint:

• The support constraint is applied to the entire motif rather a single residue/postion pair.

• The support constraint can be used to prune the search space besides preventing the generation of random artifacts.

One may argue that such a support constraint will result in the loss of some less frequent motifs that are statistically significant. We will explain its rationale from two perspectives: First, since the motif describes the phosphorylation pattern, it should be applicable to many substrates; otherwise, such pattern might be random artifact due to the limited number of known phosphorylation peptides. Second, we can use very small support threshold in the mining process to avoid missing infrequent motifs. In the extreme case, setting *s* = 1*/|P|* will guarantee the completeness. Certainly, this may result in the report of many meaningless motifs. From this viewpoint, we can regard the support threshold as a parameter for controlling the trade-off between completeness and false positive.

More importantly, the use of support constraint enables us to exploit a level-wise pruning strategy so as to reduce the search space. This idea has been widely used since the introduction of Apriori algorithm for association rule mining [4]. The application of this strategy to frequent motif finding is rather straightforward. For the sake of completeness, we will describe the mining procedure briefly.

In this paper, we focus on the discovery of pattern-based phosphorylation motifs, e.g., consensus sequences that consist of either conserved positions or wild positions (denoted by “.”) that can match any animo acids. Each motif has a single phosphorylated residue, which is denoted with a underlined character (S, T or Y).

We define the size of a motif as the number of conserved positions in that motif. We also call a motif of size *k* a *k*-motif. We define that one *k*-motif is the generalization of another motif if they have the same conserved residues at *k* positions. For instance, “D…S..P” and “D.Y.S..P” are 2-motif and 3-motif, respectively. And the first motif is a generalization of the second one.

Furthermore, we use *F_k_* to denote the set of all frequent *k*-motifs and *Z_k_* to denote the set of all *potential* frequent *k*-motifs.

**Figure 1:**
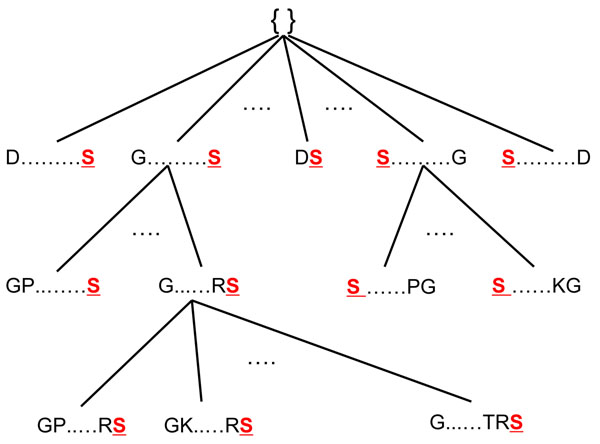
**An example search tree for browsing candidates in a level-wise manner.** We start with an empty root node and perform width-first search. Only the nodes of frequent motifs are expanded for further checking.

To find all frequent motifs, we utilize the level-wise search strategy rooted from the Apriori algorithm [4]. More precisely, the search procedure has multiple iterations to obtain the set of all frequent motifs. In the first iteration, we scan *P* to count the the number of phosphorylated peptides that match each possible 1-motif. In the subsequent *k*th iterations, we perform the following two operations:

1. Only the frequent motifs from *F_k_*_–1_ are used to generate *Z_k_* since a *k*-motif will not be frequent if one of its (*k* – 1) generalizations is infrequent. Therefore, the search space for *k*-motifs is reduced. An example search tree is given in Fig.1.

2. The set *P* is scanned to count the support of candidates in *Z_k_.* Less frequent motifs are deleted from *Z_k_* to generate *F_k_*.

We stop the search when *F_k_* is empty and return *F =* ∪*F_k_* as the final result.

### Motif-All algorithm

**Figure 2:**
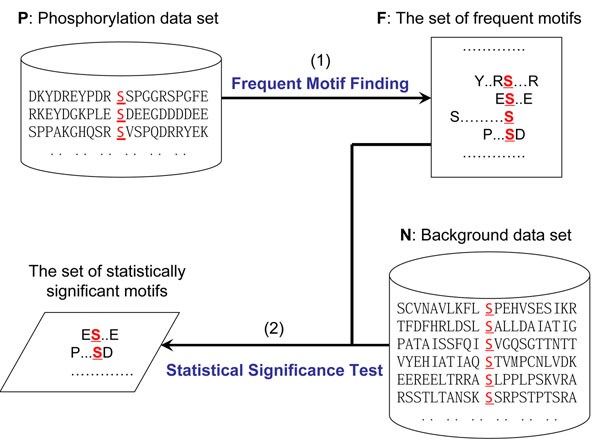
**Overview of the Motif-All algorithm**. In the first step, we find frequent motifs from *P* to reduce the number of candidate motifs. In the second step, we perform significance test to report all over-expressed frequent motifs to the user.

The Motif-All algorithm takes the support threshold *s* and the significance threshold *θ* as input to find phosphorylation motifs from two sets: a set of phosphorylated peptides *P* and a set of unphosphorylated peptides *N.* As shown in Fig.2, it consists of the following steps:

1. Finding the set of all frequent motifs *F* from *P* using the level-wise search method introduced in the previous subsection.

2. Scanning the set *N* to calculate the log odds ratio for each motif *m* ∈ *F.* Those motifs whose *p*-values are no greater than *θ* are returned to the user.

Note that the statistical evaluation of motifs can be done in various ways. Thus, we can also use other measures in Motif-All by simply replacing the log odds ratio with the new significance measure in step 2. The choice of significance evaluation measure will not change the completeness property of our algorithm.

## Results

### Data

To test the performance of Motif-All, we use the PhosPhAt database 3.0 of Arabidopsis phosphorylation sites [5,6] to construct the set of phosphorylated peptides *P.* Note that we only utilize unambiguous site identifications in the construction process. The length of each extracted peptide is 21 with a measured phosphorylated residue in the 11th position. To generate the background data set *N,* we first extract all 21-mers with a phosphorylated residue in the center position from the TAIR7 protein database. Then, we remove all peptides already in *P.* The remaining peptides form the background data.

One may worry that such background data generation procedure will disable the extraction of meaningful motifs since *N* contains many peptides that can be phosphorylated but are not identified so far. We like to point out that these potential phosphorylated peptides are overwhelmed by those truly non-phosphorylated peptides in *N*. Thus, this data generation does not change the characteristics of the background set *N*. Overall, we generate three groups of data for serine (denoted by PhAtS), threonline (denoted by PhAtT) and tyrosine (denoted by PhAtY), respectively. Their characteristics are the following:

• PhAtS: It contains 2734 foreground sequences and 982050 background sequences.

• PhAtT: It contains 415 foreground sequences and 550574 background sequences.

• PhAtY: It contains 80 foreground sequences and 304344 background sequences.

### Performance comparison

In the experiments, we compare our Motif-All algorithm against the Motif-X algorithm [2] and the MoDL algorithm [3]. Note that we didn’t include other motif finding algorithms such as TEIRESIAS [8] in the comparison. This is because these algorithms are not designed for phosphorylation motif discovery and it has been shown that MoDL [3] is superior to these methods.

**Figure 3:**
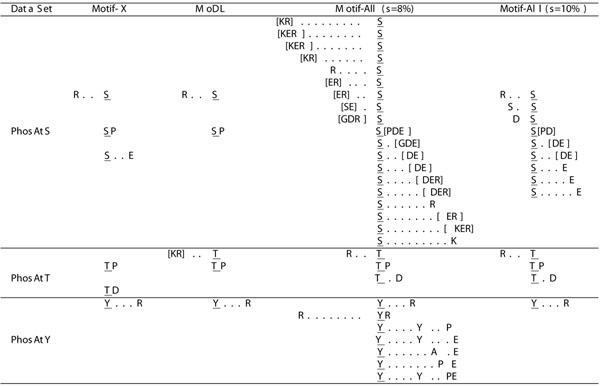
**Performance comparison using data generated from PhosPhAt database 3.0.** In Motif-All, we fix the *p*-value threshold to be 10*^-^*^6^ and report the results at the support threshold of 8% and 10%, respectively. Since it is not allowed to upload more than 10MB data to the Motif-X server, we set the “background” option of Motif-X to “IPI Arabidopsis Proteome” to mimic the background data in our local machine. As shown in [2], Motif-X is robust to the choice of background data. Therefore, it is acceptable to use such setting in the performance comparison. Since the occurrence threshold of Motif-X has a similar effect as the support constraint of Motif-All, we set this parameter to be the value that is equivalent to the support value of 8%, i.e., it is specified as 219, 33 and 6 for PhAtS, PhAtT and PhAtY, respectively. We download the Matlab codes of MoDL to perform motif discovery using its default configurations. The notation “[]” denotes a conserved position that match any letter contained in the bracket.

The results of Motif-All, Motif-X and MoDL are listed in Fig.3. Some important observations are summarized as follows.

Firstly, our algorithm is able to find more statistically significant phosphorylation motifs than existing algorithms. This is because our method has a theoretical guarantee on the completeness of results under a given parameter setting. In particular, almost all reported motifs of Motif-X and MoDL are included in the motif set of Motif-All. There are two exceptions: one is the motif “TD” detected by Motif-X and another is the motif “K . . T” found by MoDL. After checking these two motifs carefully, we found that the *p*-value of “TD” is 1*.*1 × 10^–5^ and the *p*-value of “K . . T” is 2*.*4 × 10^–3^ according to our significance test. Obviously, both motifs are not statistically significant according to the *p*-value threshold of 10^–6^. Secondly, the increase of support threshold for Motif-All will generate motif set that is very similar to that of Motif-X and MoDL. This is clearly visible in PhAtT and PhAtY. To further check if this is true, we also perform motif finding on PhAtS at the support threshold of 15%. We obtain three motifs under this setting: “R . . S”, “S . S” and “SP”. This result set is almost identical to that of Motif-X and MoDL listed in Fig.3. This means that Motif-All not only can find more useful motifs but also is capable of serving as a substitute of existing algorithms in a flexible manner.

Finally, we can generate more motifs using Motif-X by lowering the occurrence threshold. For instance, if the occurrence threshold is set to 20 on PhAtS, Motif-X will return 31 motifs. To test whether Motif-All can still find these motifs, we use an approximately equivalent setting of *s* = 1% to conduct the experiment. Totally, Motif-All detects 1153 significant motifs that include all 31 motifs reported by Motif-X. The detailed results are available at http://bioinformatics.ust.hk/MotifAll.rar.

### Effect of support constraint

**Figure 4:**
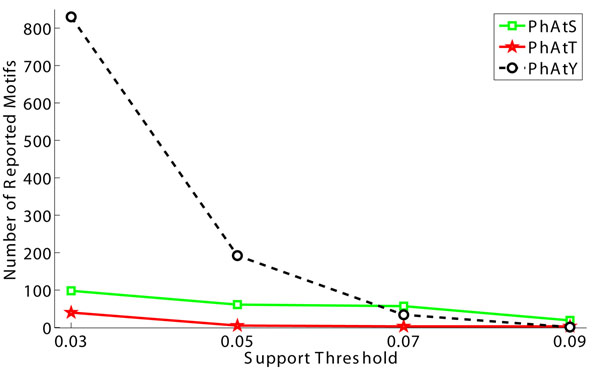
**The effect of support constraint on the number of identified motifs.** Note that the number of identified motifs may far exceed the number of phosphorylated peptides. This is because the number of potential phosphorylation motifs is 21^(^*^L^*^–1)^ – 1, where *L* is the length of each peptide.

**Figure 5:**
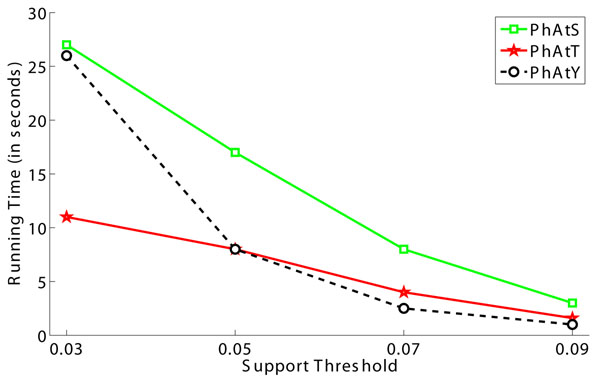
**The effect of support constraint on the running time.** Our algorithm is implemented with Java and is tested on a ThinkPad T400 notebook computer with 2.4GHz CPU and 3GB RAM.

To test the effect of support threshold, we vary this parameter from 0.03 to 0.09 and plot the corresponding numbers of discovered motifs and the running time in Fig.4 and Fig.5, respectively. Clearly, the number of identified motifs will decrease when the support threshold increases. Moreover, the use of a smaller support threshold may generate too many motifs. Therefore, it is plausible to give priority to larger support thresholds in parameter assignment.

The running efficiency test in Fig.5 shows that the increase of support threshold will lead to the decrease of running time as well. More importantly, Motif-All can finish the motif finding procedure within 30 seconds, which is faster than Motif-X and MoDL. This means Motif-All is capable of discovering motifs from large data sets very efficiently.

## Discussion

The discovery of phosphorylation motifs is a computationally challenging problem. This paper tries to resolve it through the introduction of Motif-All algorithm. Here we discuss several related problems that may need to be further investigated.

• **Problem formulation:** One critical question is how to formulate the computational problem for phosphorylation motif discovery. Currently, it is casted either as a search problem or as an optimization problem. Motif-X falls into the first category while MoDL belongs to the second category. In this paper, we opt for the first category since such formulation guarantees that each identified motif is statistically significant. However, it may report too many motifs than necessary as the statistical significance may not necessarily agrees with the biological interpretation. In this regard, optimization-based formulation has the merit of generating a concise motif set. Therefore, a better problem formulation incorporating the biological knowledge of phosphorylation is still needed.

• **Motif evaluation:** In Motif-All, we use two measures for motif assessment: statistical significance and support. Though our initial motivation of introducing the support constraint was to reduce the search space, we later found that it is also of practical importance in motif evaluation. Without the help of support constraint, it is almost impossible to obtain a concise motif set using only the significance test. From this perspective, we strongly recommend the adoption of support as one standard measure for phosphorylation motif evaluation in future studies.

Furthermore, we also need to develop new evaluation measures since the combination of significance threshold and support is still insufficient for separating true phosphorylation motifs from false ones in some cases. The general question of developing an effective evaluation measure is open and needs more investigations.

• **Motif utilization:** Experimental methods to identify phosphorylation sites are time consuming, labor intensive and expensive [9,10]. The identified motifs can be used to predict potential phosphorylation sites before biological validation. For instance, the recent released Scan-X method [11] is one such representative, which is built on Motif-X. The capability of finding more significant motifs using Motif-All makes it possible to build more accurate classifiers for better prediction.

## Conclusions

We introduced the Motif-All algorithm for finding phosphorylation motifs. Motif-All can identify all statistically significant motifs under a given parameter setting. Meanwhile, it is very fast such that it is able to find hundreds of meaningful motifs from millions of peptide sequences within one minute on a personal computer. Our experimental results show that it outperforms existing phosphorylation motif discovery algorithms.

We have shown that Motif-All is able to find more phosphorylation motifs than existing algorithms. However, it is very expensive and difficult to perform biological validation. To measure the correctness of the identified motifs that are not reported by other algorithms, one alternative strategy is to perform permutation test so as to control the false positive rate. Unfortunately, the permutation test is a very time-consuming procedure since it needs to execute Motif-All many times. To address this issue, our future work will focus on the design and implementation of fast algorithms that support large permutation test.

## Competing interests

The authors declare that they have no competing interests.

## Authors' contributions

ZH,CY and GG performed the implementations and drafted the manuscript. NL and WY conceived the study and finalized the manuscript. All authors read and approved the final manuscript.
